# Social anxiety and suicidal ideation among middle-school students in China: a mediation model of internet addiction

**DOI:** 10.3389/fpsyt.2023.1337577

**Published:** 2024-01-04

**Authors:** Yafei Tan, Jingjing Deng, Dan Zhang, Chang Peng, Anna Peng

**Affiliations:** ^1^Wuhan Children’s Hospital (Wuhan Maternal and Child Healthcare Hospital), Tongji Medical College, Huazhong University of Science & Technology, Wuhan, Hubei, China; ^2^College of Public Health and Health Professions, Hubei University of Science and Technology, Xianning, Hubei, China; ^3^School of Public Health, Tongji Medical College, Huazhong University of Science and Technology, Wuhan, Hubei, China

**Keywords:** suicidal ideation, social anxiety, internet addiction, Chinese adolescents, mediation

## Abstract

**Background:**

Suicide is a fatal public health issue for adolescents, and it is of great significance to explore the precursors of suicidal behaviors, especially suicidal ideation. However, the relationship between social anxiety and suicidal ideation and its mechanism are still unclear. The study aims to examine the association between social anxiety and suicidal ideation and the mediating effect through Internet addiction.

**Methods:**

A total of 2,278 middle-school students aged 12 to 16 years were recruited through a multistage cluster sampling method in this cross-sectional study. Logistical regression analysis and structural equation modeling (SEM) were conducted to examine the direct and indirect effects of social anxiety.

**Results:**

During the past year, 262 (11.50%) participants reported suicidal ideation. Females had a higher prevalence of suicidal ideation than males (12.9% vs. 10.0%, *p* = 0.034), and urban adolescents reported a higher prevalence than their rural counterparts (13.4% vs. 9.6%, *p* = 0.006). In the total sample, social anxiety and Internet addiction were independently associated with suicidal ideation (*p* < 0.05). In the subgroup analysis, the association between social anxiety and suicidal ideation was significant only among rural females and urban males (*p* < 0.05). SEM demonstrated that social anxiety had direct and indirect effects on suicidal ideation, and Internet addiction partially mediated the relationship, with a mediating ratio of 30.53%. The partial mediating effect was also significant only in rural females and urban males.

**Conclusion:**

Adolescents may overuse the Internet to cope with social anxiety and further have suicidal ideation. Limiting Internet use and improving interpersonal skills in real life may be efficient for suicide prevention. In addition, targeted interventions should be tailored by different sexes across urban and rural regions.

## 1 Introduction

Suicide is a serious public concern worldwide and is the fourth leading cause of death among youth aged 15 to 29 years (1). Adolescence has been identified as a high-risk period of onset of suicidal thoughts and behaviors, including suicidal ideation, suicide plans, suicide attempts, and suicide death (2). Previous suicidal ideation is a strong predictor of subsequent suicide attempts and suicide death (3, 4), suggesting that suicidal ideation should be the first indicator and major maker when screening suicidal risk. In China, the prevalence of suicidal ideation in adolescents during the last year ranges from 13.7 to 16.0% (5, 6). To prevent subsequent suicide attempts and suicide dearth, it is urgent to identify risk factors for suicidal ideation and to develop targeted prevention strategies for suicidal ideation (7). The literature has found that some psychosocial factors are significantly related to suicidal ideation in youth, such as depression (8), anxiety (9), substance abuse (10, 11), sleep problems (12, 13), childhood maltreatment (14, 15), and bullying victimization (16, 17).

### 1.1 Social anxiety and suicidal ideation

Apart from the risk factors mentioned above, researchers have begun to assess the relationship between social anxiety and suicidality for adolescents and young people (7, 18). Social anxiety, also known as social phobia, refers to a persistent fear of being exposed to social or professional situations where people might feel they are being observed by others (19). Additionally, social anxiety is marked by a fear of engaging in embarrassing behaviors or having negative evaluations by others (19, 20). An individual exposed to such situations will generally suffer or undergo intense anxiety. In return, anxiety can severely impair the development of social functions and inhibit them from actively engaging in their social relationships (21). Self-reported symptoms of social anxiety increase from childhood and appear to peak in adolescence, and the prevalence of social anxiety ranges from 4.0 to 15.3% among adolescents and young adults (21, 22).

Social anxiety is often associated with comorbidities such as depression, substance abuse, and suicidal ideation (23, 24). For example, a recent study indicated that a higher level of social anxiety was related to an increased risk of current suicidal ideation in 408 Korean medical students (18). Similarly, clinical research based on 144 adolescents aged 12 to 15 years in the United States found that social anxiety at baseline had positive direct and indirect effects on subsequent suicidal ideation through loneliness (7). However, to date, little is known about the possible link between social anxiety and suicidality in Chinese adolescents, which deserves further investigation since cultural background in different countries may influence the association.

### 1.2 The potential mediation of internet addiction

In addition to social anxiety, Internet addiction has been examined as another important risk factor for adolescents’ suicidality (25–27). Internet addiction, also known as problematic Internet use (PIU), is defined as overuse of the Internet to some extent where it disrupts daily life of individuals and they lose control over Internet use that replaces usual and expected social, educational and/or work, relationship, and family activities, leads to clinically significant impairment or suffering, such as preoccupation, withdrawal, tolerance, failed attempts to control, loss of interest, deception, escape, and functional impairment (28–30). Although Internet addiction will occur at any age, adolescence is the highest-risk period. Adolescents can promptly accept and indulge in the latest technologies as well as the least social media tools (31). Meanwhile, they are more vulnerable to the potential adverse effects of Internet use than other groups (32). For example, Huang et al. (33) found that Internet addiction was independently correlated with suicidal ideation in 12,507 Chinese adolescents (33). Another previous study revealed that adolescents with Internet addiction had 1.76 times the odds ratio (OR) of suicidal ideation, 2.13 times the odds ratio of suicide plans, and 3.16 times the odds ratio of suicide attempts compared to adolescents without Internet addiction in rural China (34).

On the other hand, emerging research has found a positive correlation between social anxiety and Internet addiction (21, 35), and social anxiety is a significant predictor of Internet addiction (36, 37). According to the cognitive-behavioral model (38), social anxiety could be viewed as a distal antecedent of Internet addiction. For instance, individuals with social anxiety generally have frustrated and failed interpersonal relationships due to their deficiency of social skills or even their incompetence and worthlessness. These individuals may be more likely to overuse the Internet due to the immediate and positive effect of online activities on alleviating pressure and distress in the real world (39).

Given the positive correlation between social anxiety and suicidal ideation, Internet addiction and suicidal ideation, and social anxiety and Internet addiction, it is reasonable to assume the indirect effect of social anxiety on suicidal ideation through the mediating role of Internet addiction. A previous study conducted in 5,366 adolescents aged 12 to 18 years from six Asian countries has shown that Internet addiction can mediate the relationship between social anxiety and poor psychosocial well-being in China and Malaysia (39). However, psychosocial well-being did not include suicidal thoughts or behaviors. Therefore, it is worth exploring whether Internet addiction can also play a mediating role in the relationship between social anxiety and suicidal ideation. The findings will help better understand the development of suicidality and largely benefit the efficient prevention of suicide.

### 1.3 The difference in sex and residence

Much existing evidence supports that sex is independently associated with social anxiety, Internet addiction, and suicidal ideation (7). For instance, Jin et al. (22) found that the level of social anxiety was significantly higher in females than in males (22). Li et al. (40) found that the prevalence of Internet addiction among males was significantly higher than among females (13.7% vs. 6.6%) (40). In addition, many empirical studies believe that females are more likely to report suicidal ideation than males (5, 41–43). Therefore, it is necessary to examine sex differences when exploring the mediation of Internet addiction between social anxiety and suicidality.

Similarly, an increasing number of researchers have noted that the place of residence is also an influencing factor of the three variables mentioned above. Specifically, compared to rural adolescents, urban adolescents may have a higher level of social anxiety (44) and a higher prevalence of Internet addiction (40). However, according to two sample-based mortality surveillance systems of suicide rates in China, the incidence rate ratio (IRR) of suicide rates in rural areas (IRR = 1.93, 95% CI: 1.84–2.03) is higher than that in urban areas (45). To date, few studies have examined whether the prevalence and risk odds of suicidal ideation differ between urban and rural adolescents (46). Thus, the underlying effect of residence on the relationship of social anxiety, Internet addiction, and suicidal ideation warrants further exploration.

### 1.4 The current study

To the best of our knowledge, the mediating role of Internet addiction between social anxiety and suicidal ideation has not been explored. The aims of this study are as follows. First, we will assess whether social anxiety and Internet addiction are independently associated with suicidal ideation by logistical regression analysis. Second, we will examine the indirect effect of social anxiety on suicidal ideation with the mediating role of Internet addiction through structural equation modeling (SEM) analysis. Third, we will further explore whether the relationship between social anxiety, Internet addiction, and suicidal ideation differs by sex and place of residence through subgroup analysis.

## 2 Methods

### 2.1 Study participants and data collection

A multistage cluster sampling method was used in this cross-sectional study in Xiamen City. In stage 1, we divided the city into two geographic areas (urban and rural). In stage 2, three middle schools were randomly selected in each area. In stage 3, two or three classes from each grade (7^th^ to 9^th^) were selected using random digits in all selected schools based on enrollment size. Finally, 46 classes were selected and all students in the selected classes were invited to participate in the survey voluntarily and then completed a self-report questionnaire. Of 2,400 students who submitted the questionnaire, 122 questionnaires were excluded because more than 15% of the data were missing. Finally, 2,278 participants’ questionnaires were qualified and the actual response rate of the current study was 94.92% (2,278/2400).

### 2.2 Measures

#### 2.2.1 Social anxiety

The Social Anxiety subscale of the Self-Consciousness Scale (SASS) was designed to measure some domains of social anxiety, such as subjective anxiety and verbal and behavioral difficulties (47). Every item was rated on a 5-point Likert scale (from 0 = “strongly disagree” to 4 = “totally agree”), and the total core ranged from 0 to 24 points. A higher total SASS score indicated a higher degree of social anxiety. The Chinese version of SASS had good reliability and validity in some previous studies (22, 44), with a Cronbach’s alpha of 0.76 in the current study.

#### 2.2.2 Internet addiction

Internet addiction was measured using the Chinese version of the Young’s Internet Addiction Test (IAT) (30). The IAT comprised 20 items rated on a 5-point Likert scale (from 1 = “not at all” to 5 = “always”). The total IAT score ranges from 20 to 100, and a higher total score suggests a greater tendency to Internet addiction (48). The IAT has been validated in Chinese adolescents with satisfactory psychometric properties (49). The Cronbach’s alpha of the IAT in this study was 0.905.

#### 2.2.3 Suicidal ideation

Suicidal ideation was assessed using a related item from the Global School-Based Student Health Survey, which refers to “Have you ever had serious thoughts of killing yourself during the past year?” The responses to the questions were dichotomized as no (0 times) vs. yes (1 or more times) (5, 18).

#### 2.2.4 Social-demographic characteristics

Social-demographic variables included sex (1 = male, 2 = female), place of residence (1 = urban, 2 = rural), age, grade (7^th^, 8^th^ and 9^th^), parental marital status (1 = married, 2 = divorced or other), only child (0 = no, 1 = yes), parents’ education (referring to a higher education level of two parents, 1 = primary school or less, 2 = middle high school, 3 = senior high school, 4 = college or more), family income (family income per year in RMB: 1 = less than 20,000, 2 = 20,001–69,999, 3 = more than 70,000), and academic performance (referring to perceived academic performance in the class, 1 = good, 2 = moderate, 3 = poor, 4 = other).

### 2.3 Data analysis

First, the sociodemographic characteristics of the participants and the prevalence of suicidal ideation were summarized by descriptive statistics. Continuous variables were described by mean (SD), such as age and IAT score. Second, the chi-square test was used to compare the prevalence of suicidal ideation in different categorical variables. Student’s *t* test was used to compare the mean SASS and IAT scores between participants with and without suicidal ideation. One-way ANOVA was used to compare the mean SASS and IAT scores between the four groups of sex × place of residence (rural male, rural female, urban male, and urban female). Third, to examine the independent effects of social anxiety and Internet addiction on suicidal ideation (0 = No, 1 = Yes), binary logistic regression analysis was adopted to assess odds ratios (ORs) and 95% confidence intervals (95% CIs) with SASS and IAT scores as two independent variables. In addition, we included all sociodemographic characteristics as confounding variables. Fourth, a subgroup analysis was conducted to assess the potential role of sex × place of residence, and a set of binary logistic regression analyses was performed among rural males, rural females, urban males, and urban females separately. The significance level was set at *p* < 0.05, and all tests were two-sided. All data were analyzed with IBM SPSS, version 26.0.

Fifth, we performed structural equation modeling (SEM) to evaluate the mediating effects of Internet addiction (IAT score) on the relationship between social anxiety (SASS score) and suicidal ideation. In model 1, we ran SEM with the total sample. In models 2 to 5, we ran SEM among rural males, rural females, urban males, and urban females separately to assess the influence of sex × place of residence in the mediation model. All models were adjusted for variables that had significant associations with suicidal ideation in the binary logistic regression analysis. All models of SEM were analyzed using IBM SPSS Amos 21.0.

## 3 Results

### 3.1 Sample information

Among the 2,278 participants, 1,105 were males (48.5%) and 1,173 were from urban areas (51.5%). Half of the participants (1,140, 50.0%) were from rural regions, and over three quarters (1745, 76.6%) were from families with multiple children. The mean age (SD) was 13.71 (1.05). Other characteristics of the sample are depicted in Table 1. In addition, the prevalence of suicidal ideation was 11.50% (262/2278) during the past year.

**Table 1:** Characteristics of participants and prevalence of suicidal ideation.

Variables	Total (*n* = 2,278)	Suicidal ideation		
		Yes (*n* = 262)	No (*n* = 2016)	*p* value
Sex				0.034
Male	1,105 (48.5)	111 (10.0)	994 (90.0)	
Female	1,173 (51.5)	151 (12.9)	1,022 (87.1)	
Place of residence				0.006
Rural	1,440 (50.0)	110 (9.6)	1,030 (90.4)	
Urban	1,138 (50.0)	152 (13.4)	986 (86.6)	
Grade				0.927
7	741 (32.5)	88 (11.9)	653 (88.1)	
8	760 (33.4)	86 (11.3)	674 (88.7)	
9	777 (34.1)	88 (11.3)	689 (88.7)	
Parental marital status				< 0.001
Married	2,130 (93.5)	231 (10.8)	1899 (89.2)	
Divorced or other	148 (6.5)	31 (20.9)	117 (79.1)	
Only child				0.299
No	1745 (76.6)	194 (11.1)	1,551 (88.9)	
Yes	533 (23.4)	68 (12.8)	465 (87.2)	
Parents’ education				0.083
Primary school or less	328 (14.4)	47 (14.3)	281 (85.7)	
Middle high school	1,104 (48.5)	109 (9.9)	995 (90.1)	
Senior high school	571 (25.1)	69 (12.1)	502 (87.9)	
College or more	275 (12.1)	37 (13.5)	238 (86.5)	
Family income (RMB)				0.010
Less than 20,000	799 (35.1)	85 (10.6)	714 (89.4)	
20,001–69,999	991 (43.5)	102 (10.3)	889 (89.7)	
More than 70,000	488 (21.4)	75 (15.4)	413 (84.6)	
Academic performance				0.752
Good	748 (32.8)	87 (11.6)	661 (88.4)	
Moderate	497 (21.8)	59 (11.9)	438 (88.1)	
Poor	723 (31.7)	86 (11.9)	637 (88.1)	
Other	310 (13.6)	30 (9.7)	280 (90.3)	
Age, M (SD)	13.71 (1.05)	13.69 (0.99)	13.71 (1.05)	0.799
SASS, M (SD)	5.99 (6.25)	8.26 (6.73)	5.70 (6.13)	< 0.001
IAT, M (SD)	32.48 (12.12)	39.96 (14.22)	31.51 (11.48)	< 0.001

SASS: the Social Anxiety subscale of the Self-Consciousness Scale, IAT: Young’s Internet Addiction Test.

### 3.2 Univariate analysis of suicidal ideation

Compared to participants without suicidal ideation, those participants with suicidal ideation reported a higher level of social anxiety [8.26 (6.73) vs. 5.70 (6.13), *p* < 0.001)] as well as Internet addiction [39.96 (14.22) vs. 31.51 (11.48), *p* < 0.001)]. Females reported more suicidal ideation than males (12.9% vs. 10.0%, *p* = 0.034). Urban adolescents reported more suicidal ideation than their rural counterparts (13.4% vs. 9.6%, *p* = 0.006). In addition, the prevalence of suicidal ideation was significantly different in terms of parental marital status and family income (*p* < 0.05) (Table 1).

With regard to the interaction of sex × place of residence, the prevalence of suicidal ideation from high to low was 14.6% (urban female), 12.1% (urban male), rural female (11.2%), and 7.9% (rural male). In a pairwise comparison, the prevalence of suicidal ideation among rural males was significantly lower than among urban males and females (*p* < 0.05). Additionally, SASS and Internet addiction scores were significantly different among participants across sex × place of residence (Table 2).

**Table 2:** The prevalence of suicidal ideation and the score difference of SASS and IAT among participants of sex × place of residence.

Variables	Sex× Place of residence					
	Rural Male (*n* = 535)	Rural Female (*n* = 605)	Urban Male (*n* = 570)	Urban Female (*n* = 568)	*p value*	Pairwise comparison
Suicidal ideation						
No	493 (92.1)	537 (88.8)	501 (87.9)	485 (85.4)		
Yes	42 (7.9)	68 (11.2)	69 (12.1)	83 (14.6)	0.005	c, d > a
SASS, M (SD)	6.68 (6.43)	7.21 (6.28)	4.87 (6.10)	5.19 (5.88)	< 0.001	a, b > c, d
IAT, M (SD)	33.30 (12.98)	30.25 (10.77)	34.51 (13.08)	32.07 (11.23)	< 0.001	a, c > d > b

a: Rural Male, b: Rural Female, c: Urban Male, d: Urban Female in pairwise comparison.

SASS: the Social Anxiety subscale of the Self-Consciousness Scale, IAT: Young’s Internet Addiction Test.

### 3.3 Logistic regression analysis of suicidal ideation

After adjusting for covariates, the SASS score (OR = 1.049, 95% CI = 1.028–1.071, *p* < 0.001) and the IAT score (OR = 1.042, 95% CI = 1.032–1.052, *p* < 0.001) were significantly associated with a higher odds ratio of suicidal ideation. Compared to males, females had higher odds of suicidal ideation (OR = 1.586, 95% CI = 1.199–2.097, *p* < 0.01). Urban participants had higher risk odds of suicidal ideation than rural participants (OR = 1.513, 95% CI = 1.077–2.127, *p* < 0.05). In addition, parental marital status was significantly associated with suicidal ideation (Table 3).

**Table 3:** Binary logistic regression of suicidal ideation among participants by sex× place of residence[OR (95% CI)].

Variables	Total (n = 2,278)	Sex × Place of residence			
		Rural male (n = 535)	Rural female (n = 605)	Urban male (n = 570)	Urban female (n = 568)
SASS (1 score increase)	1.049 (1.028–1.071)^***^	1.050 (0.999–1.103)	1.076 (1.032–1.123)^**^	1.061 (1.020–1.104)^**^	1.011 (0.970–1.055)
IAT (1 score increase)	1.042 (1.032–1.052)^***^	1.029 (1.007–1.051)^*^	1.047 (1.026–1.070)^***^	1.037 (1.018–1.057)^***^	1.052 (1.031–1.073)^***^
Sex					
Male	1.000	NA	NA	NA	NA
Female	1.586 (1.199–2.097)^**^	NA	NA	NA	NA
Place of residence					
Rural	1.000	NA	NA	NA	NA
Urban	1.513 (1.077–2.127)^*^	NA	NA	NA	NA
Grade					
7	1.000	1.000	1.000	1.000	1.000
8	0.792 (0.525–1.194)	1.615 (0.533–4.891)	0.730 (0.296–1.801)	0.998 (0.457–2.181)	0.442 (0.211–0.924)^*^
9	0.733 (0.412–1.304)	3.483 (0.895–13.549)	1.137 (0.351–3.680)	0.824 (0.256–2.648)	0.141 (0.044–0.454)^**^
Parental marital status					
Married	1.000	1.000	1.000	1.000	1.000
Divorce or other	1.995 (1.268–3.140)^**^	1.797 (0.486–6.638)	4.140 (1.502–11.412)^**^	1.411 (0.557–3.574)	2.467 (1.129–5.391)^*^
Parents’ education					
Primary school or less	1.000	1.000	1.000	1.000	1.000
Middle high school	0.684 (0.462–1.011)	0.975 (0.447–2.125)	0.497 (0.265–0.932)^*^	0.427 (0.147–1.244)	0.889 (0.315–2.508)
Senior high school	0.733 (0.464–1.158)	1.509 (0.525–4.336)	0.380 (0.129–1.120)	0.464 (0.164–1.311)	0.884 (0.312–2.504)
College or more	0.764 (0.440–1.326)	0.989 (0.675–1.983)	2.079 (0.597–7.243)	0.403 (0.127–1.273)	0.972 (0.314–3.004)
Family income (RMB)					
Less than 20,000	1.000	1.000	1.000	1.000	1.000
20,001–69,999	0.984 (0.712–1.360)	0.945 (0.452–1.977)	1.110 (0.592–2.084)	0.559 (0.292–1.069)	1.747 (0.871–3.503)
More than 70,000	1.394 (0.961–2.023)	0.936 (0.357–2.455)	2.897 (1.303–6.439)^**^	0.760 (0.378–1.529)	2.350 (1.123–4.918)^*^
Age (1 year increase)	1.092 (0.865–1.379)	0.667 (0.386–1.152)	0.995 (0.618–1.605)	0.958 (0.606–1.516)	2.053 (1.286–3.280)^*^

SASS: the Social Anxiety subscale of the Self-Consciousness Scale, IAT: Young’s Internet Addiction Test.

Only child and academic performance were not significant in all models (*p* > 0.05).

***p < 0.001, **p < 0.01, *p < 0.05.

In the subgroup analysis, the IAT score was positively related to the higher risks of suicidal ideation for all groups of sex × place of residence, while the SASS score was significantly associated with the higher risks of suicidal ideation only for rural females (OR = 1.076, 95% CI = 1.032–1.123, *p* < 0.01) and urban males (OR = 1.061, 95% CI = 1.020–1.104, *p* < 0.01) (Table 3).

### 3.4 The mediating effect of internet addiction between social anxiety and suicidal ideation

**Figure 1:**
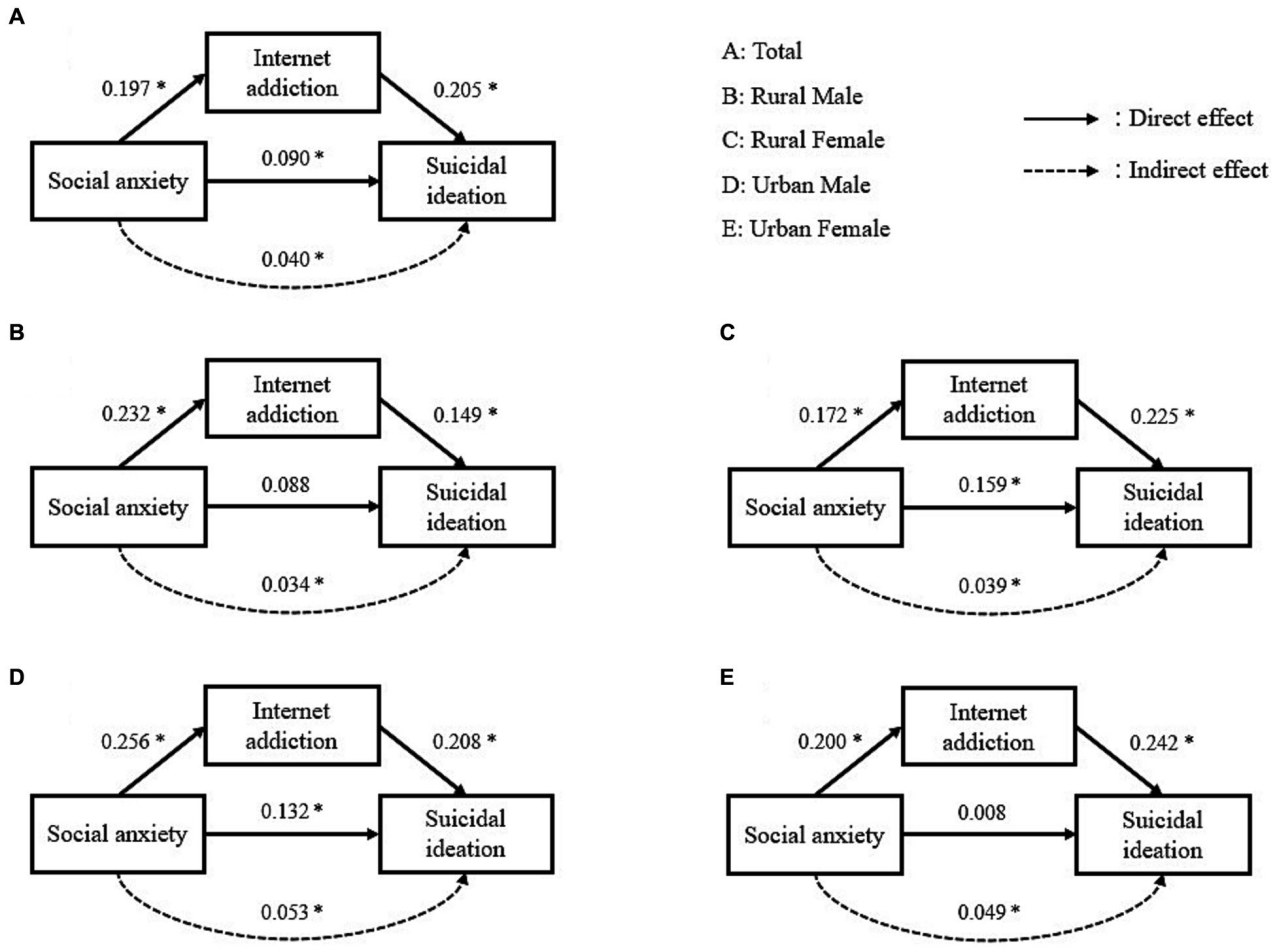
Structural equation modelling depicting direct and indirect effects (adjusted β coefficients) of social anxiety on suicidal ideation. All models adjusted for significant covariates in the logistic regression analysis of suicidal ideation, including age, grade, parental marital status, parents’ education, and family income. ^*^p < 0.05.

After controlling for covariates, there were direct effects of the SASS score (*β* = 0.090, 95% CI = 0.045–0.139, *p* < 0.05) and the IAT score (*β* = 0.205, 95% CI = 0.155–0.253, *p* < 0.05) on suicidal ideation. The total effect of the SASS score on suicidal ideation was 0.131 (95% CI = 0.085–0.177, *p* < 0.05), and the indirect effect was 0.040 (95% CI = 0.028–0.055). The mediation ratio was 30.53% (Table 4). For males and females in urban and rural areas, the direct effects of the SASS score on suicidal ideation were significant only for rural females and urban males (*p* < 0.05). The total effect of the SASS score on suicidal ideation was not significant for urban females (*p* > 0.05). The mediation ratios of the IAT score between the SASS score and suicidal ideation from high to low were 87.50% (urban female), 28.65% (urban male), 27.64% (rural male), and 19.70% (rural female), respectively (Figure 1). These results indicate that the IAT score plays a partial mediating role between the SASS score and suicidal ideation among the total sample, rural females, and urban males, while the IAT score plays a total mediating role among rural males.

**Table 4:** Mediating effect of IAT between SASS and suicidal ideation [Standardized β estimate (95% CI)].

Sample groups	Direct effect			Indirect effect	Total effect	Mediating ratio,%
	SASS→ Suicidal ideation	SASS→IAT	IAT → Suicidal ideation			
Total	0.090 (0.045–0.139) ^*^	0.197 (0.152–0.246) ^*^	0.205 (0.155–0.253) ^*^	0.040 (0.028–0.055) ^*^	0.131 (0.085–0.177) ^*^	30.53
Rural male	0.088 (−0.009–0.179)	0.232 (0.162–0.319) ^*^	0.149 (0.056–0.251) ^*^	0.034 (0.013–0.066) ^*^	0.123 (0.021–0.208) ^*^	27.64
Rural female	0.159 (0.069–0.232) ^*^	0.172 (0.080–0.270) ^*^	0.225 (0.129–0.347) ^*^	0.039 (0.016–0.076) ^*^	0.198 (0.106–0.274) ^*^	19.70
Urban male	0.132 (0.043–0.230) ^*^	0.256 (0.164–0.345) ^*^	0.208 (0.114–0.304) ^*^	0.053 (0.026–0.086) ^*^	0.185 (0.092–0.279) ^*^	28.65
Urban female	0.008 (−0.085–0.103)	0.200 (0.108–0.278) ^*^	0.242 (0.126–0.342) ^*^	0.049 (0.025–0.075) ^*^	0.056 (−0.041–0.156)	87.50

SASS: the Social Anxiety subscale of the Self-Consciousness Scale, IAT: Young’s Internet Addiction Test.

Adjusted for age, grade, parental marital status, parents’ education, and family income.

*p < 0.05.

## 4 Discussion

This is the first study to explore the direct and indirect effects of social anxiety on suicidal ideation through the mediating role of Internet addiction among Chinese middle-school students. There are several major findings. First, both social anxiety and Internet addiction are independently associated with the risk of suicidal ideation. Second, Internet addiction partially mediates the association between social anxiety and suicidal ideation. Third, the relationship between social anxiety, Internet addiction, and suicidal ideation is distinct and unique between males and females in rural and urban areas. These findings expand our knowledge of the development of suicidality and help educators, clinicians, and policy makers develop efficient and targeted prevention strategies for suicide in adolescents.

### 4.1 The prevalence of suicidal ideation

The prevalence of suicidal ideation among Chinese middle-school students was 11.5% in the current study which is slightly lower than in some previous studies conducted in China ((5); C. (6)). A possible reason for the different prevalence of suicidal ideation in different studies is the assessment method. In our study only used one item to assess suicidal ideation, which may not be able to fully assess suicidal ideation among students. Consistent with most previous studies (41–43), our results support that female adolescents have a higher prevalence and risk of suicidal ideation than male adolescents. Additionally, participants living in urban areas reported higher prevalence and risk odds of suicidal ideation than participants in rural areas. Taken together, we can conclude that urban females have the highest prevalence of suicidal ideation, while rural males have the lowest prevalence in the current study. The underlying reason for this finding is that females are more likely to suffer from depression, self-harm behaviors, and living in urban areas may face greater academic competition and social stressors in Chinese adolescents (41). Since it is a new finding in the field and we have a limited sample in the study, more future surveys with representative samples are supposed to understand why suicidality is distinct and unique by sex and place of residence.

### 4.2 Association between social anxiety and suicidal ideation

In line with some previous studies (7, 18), our results demonstrate that social anxiety is independently related to suicidal ideation in Chinese adolescents after controlling for covariates. Adolescence is an important developmental period in which adolescents become more sensitive to their social relationships. However, social anxiety can cause significant mental distress and multiple impairments in social, daily routine, academic, and family functioning. Given that social anxiety is characterized by social withdrawal and deficits in social skills, it can obstruct this developmental period and further increase the risks of isolation, hopelessness, and suicidality among adolescents (7, 50). Furthermore, it is surprising to note that the association between social anxiety and suicidal ideation is significant only in rural females and urban males but is insignificant in rural males and urban females. The underlying cause of these findings was unclear. The new findings broaden the literature in the field and have practical implications for intervention strategies for suicide risk in populations with different demographic characteristics. At the same time, future research should further verify the results and explore the underlying mechanism.

### 4.3 The mediating role of internet addiction

Extending to the literature, our results first find that Internet addiction can partially mediate the relationship between social anxiety and suicidal ideation. This finding is consistent with a previous study (39), which has empirically examined the mediation model of Internet addiction between social anxiety and psychosocial problems. This proposed mediation model falls under the self-medication hypothesis that individuals with social anxiety will attempt to alleviate their mental distress through excessive Internet use (51). Under this model, Internet addiction emerges as a kind of ‘self-treatment’ in individuals who try to eliminate their social anxiety. When they have a strong fear of embarrassment in social situations or have negative evaluations from others, Internet-mediated activity may become particularly attractive since it offers greater tolerance and anonymity (37, 52). At the same time, Internet use as a coping strategy could deprive individuals from real-world social relationships and may lead to more detrimental mental outcomes when the underlying social anxiety is unknot treated. In this case, Internet addiction may become more heavy and finally become more severe mental health outcomes, such as desperation of reality and suicidal thoughts and behaviors (39).

On the other hand, interpersonal theory suggests that social anxiety can worsen attachment, interpersonal security, and self-esteem (53). However, perceived confidence and comfort can be met although Internet activity, such as online gaming or virtual communication. To compensate for feelings of inadequate social support and interpersonal insecurity, adolescents attempt to increase their self-esteem by approval from others online or by achievements in online gaming (54). However, these Internet approaches can hardly eliminate their distress and anxiety. In contrast, the combination of real-world rejection and online disappointment can further aggravate feelings of hopelessness and even suicidal ideation. There, the risk of suicidal ideation tends to be higher when adolescents have problematic Internet use, particularly in adolescents with social anxiety (54).

### 4.4 Implications

The findings of the current study have some theoretical implications for the development of suicidality among adolescents, as well as practical implications for suicide prevention. First, this study revealed that social anxiety could increase the risk of adolescents’ suicidal ideation. Clinicians involved in the assessment of suspected cases of suicidality may wish to evaluate their levels of social anxiety. Meanwhile, promoting social activities for children and adolescents in school or the community may be a promising approach to enhance their personal worth and reduce suicidal thoughts, such as participating in team sports with classmates and helping people in trouble with family members. Second, the results indicate that Internet addiction may be one mechanism through which adolescents’ social anxiety increases the risk of suicidal ideation. This finding has extended the theory of self-medication to include online activities to handle developmental stressors (51). Therefore, Internet addiction assessment is necessary among adolescents with social anxiety. Furthermore, effective interventions should be conducted in adolescents with Internet addiction to weaken the link between social anxiety and suicidality. However, since the Internet is an essential tool or an intimate partner in the daily life of youth, total prohibition or aggressive limitation of the Internet might not be a practical and efficient policy. Therefore, more efforts are needed to encourage parents and teachers to develop targeted strategies and methods to regulate Internet use in children and adolescents (54). Third, as our results suggest that the relationship between social anxiety, Internet addiction, and suicidal ideation differs by sex and residence, a targeted intervention of suicidality should be implemented. For instance, more attention should be given to rural females and urban males since the direct and indirect effects of social anxiety on suicidal ideation are significant among them.

### 4.5 Limitations

Despite the theoretical and practical implications of the current study, some limitations should be noted. First, as a cross-sectional study design, it is not possible to determine the causality between social anxiety and suicidal ideation. A longitudinal study is warranted to confirm the causal relationship of the variables in the study. Second, since the participants in our study were from one city in China, the sample may hardly represent all Chinese adolescents. Although we adopted a random sampling method and the response rate was good, the findings could not be generalized to other populations. In the next step, we will verify the results of the present study based on a large sample of adolescents using multicenter random sampling in China. Third, given the low prevalence of suicide attempts in previous studies (41, 55) and the limited sample size in our survey, we only paid attention to suicidal ideation in the current study. Furthermore, other factors that could contribute to suicidal ideation, such as school performance, depression, and bipolar disorder, which were common among teenagers, were not considered in this study. More large researches are needed to examine whether adolescents’ social anxiety is related to suicide ideation or attempts in the future.

## 5 Conclusion

Our results indicate that a higher level of social anxiety is associated with an increased risk of suicidal ideation among Chinese middle-school students. Moreover, Internet addiction partially mediates the effect of social anxiety on suicidal ideation. In addition, the relationship of social anxiety, Internet addiction, and suicidal ideation may differ between males and females across rural and urban areas. On the basis of these findings, promoting social interaction and reducing social anxiety may contribute to reducing the risk of suicidality. In addition, regulating Internet use and avoiding Internet addiction could be part of the treatment of social anxiety and suicidal ideation. Furthermore, suicide prevention strategies should be tailored by sex and place of residence, and particular attention should be paid to rural females and urban males with social anxiety and Internet addiction.

## Data availability statement

The raw data supporting the conclusions of this article will be made available by the authors, without undue reservation.

## Ethics statement

The studies involving humans were approved by Institutional Review Board of Wuhan Children’s Hospital. The studies were conducted in accordance with the local legislation and institutional requirements. Written informed consent for participation in this study was provided by the participants' legal guardians/next of kin.

## Author contributions

YT: Formal Analysis, Writing – original draft, Writing – review & editing. JD: Data curation, Investigation, Writing – review & editing. DZ: Data curation, Investigation, Writing – review & editing. CP: Methodology, Validation, Writing – review & editing. AP: Conceptualization, Project administration, Supervision, Writing – review & editing.
